# Is Replication the Gold Standard for Validating Genome-Wide Association Findings?

**DOI:** 10.1371/journal.pone.0004037

**Published:** 2008-12-29

**Authors:** Yong-Jun Liu, Christopher J. Papasian, Jian-Feng Liu, James Hamilton, Hong-Wen Deng

**Affiliations:** 1 School of Medicine, University of Missouri - Kansas City, Kansas City, Missouri, United States of America; 2 The Key Laboratory of Biomedical Information Engineering of Ministry of Education, and Institute of Molecular Genetics, School of Life Science and Technology, Xi'an Jiaotong University, Xi'an, Shaanxi, People's Republic of China; 3 Laboratory of Molecular and Statistical Genetics, College of Life Sciences Hunan Normal University, Changsha, Hunan, People's Republic of China; Copenhagen University Hospital, Denmark

## Abstract

With the advent of genome-wide association (GWA) studies, researchers are hoping that reliable genetic association of common human complex diseases/traits can be detected. Currently, there is an increasing enthusiasm about GWA and a number of GWA studies have been published. In the field a common practice is that replication should be used as the gold standard to validate an association finding. In this article, based on empirical and theoretical data, we emphasize that replication of GWA findings can be quite difficult, and should not always be expected, even when true variants are identified. The probability of replication becomes smaller with the increasing number of independent GWA studies if the power of individual replication studies is less than 100% (which is usually the case), and even a finding that is replicated may not necessarily be true. We argue that the field may have unreasonably high expectations on success of replication. We also wish to raise the question whether it is sufficient or necessary to treat replication as the ultimate and gold standard for defining true variants. We finally discuss the usefulness of integrating evidence from multiple levels/sources such as genetic epidemiological studies (at the DNA level), gene expression studies (at the RNA level), proteomics (at the protein level), and follow-up molecular and cellular studies for eventual validation and illumination of the functional relevance of the genes uncovered.

## Introduction

With the advent of genome-wide association (GWA) studies, it is anticipated that major susceptibility variants for common human diseases/traits can be detected [1]. With successful application of GWA studies first appearing in 2005, to date more than 190 GWA studies have been published, reporting more than 410 SNPs showing strong evidences of association with various human complex diseases/traits, such as obesity, diabetes, coronary heart diseases, asthma, cancers, mental illness, and osteoporosis (a catalog of published GWA studies is summarized at the National Human Genome Research Institute (NHGRI) website at www.genome.gov/26525384). Some GWA findings were confirmed in subsequent independent replication studies. With the belief that replication should be used as a gold standard in high quality publications, the NCI-NHGRI Working Group proposed suggestions in the June 2007 issue of *Nature* on what constitutes replication of a genotype-phenotype association, and how best to achieve this [2]. A commonly used review criterion for a GWA study is whether it is accompanied by internal (e.g., within consortium) or external (independent studies by other research groups in different populations) replication evidence.

In this article, based on empirical and theoretical data, we show that, like conventional linkage scan and candidate gene association studies, GWA results are *also* going to be very difficult to replicate even for powerful, well-designed studies. We demonstrate that 1) the probability of replication becomes smaller as the number of independent GWA studies increases if the power of individual replication studies is less than 100% (which is usually the case), and 2) statistically replicated findings are not necessarily true, although replication lessens the likelihood of the initial finding being false. We question whether it is necessary or sufficient to regard replication as the gold standard for defining a true susceptibility variant. We lastly discuss the usefulness of integrating evidence from multiple level/sources such as genetic epidemiology, functional genomics, proteomics, and molecular and cellular functional studies.

## Results

### 1) GWA Results Are Inherently Difficult to Replicate

In GWA studies, a finding is considered to be “replicated” if and only if there is an initial finding which did appropriately control for multiple testing with strong control of the family-wise type I error rate (FWER) and in a second independent sample this finding is re-detected in a confirmatory hypothesis testing setting [2]. In the following we show that the probability of replicating GWA results is inherently low. We also discuss some confounding factors that may further exacerbate the situation.

#### a) The probability of replicating GWA results is inherently low

For simplicity, we assume an ideal situation in which a gene-phenotype association exists and is identified by the initial GWA. We then examine the probability of replicating the association under three slightly different scenarios: 1) the initial GWA findings are followed by several subsequent independent replication studies (either GWA or focused regional analyses); 2) two or more independent GWA studies are conducted simultaneously; and 3) a mixture of *N* GWA (*N*<3–4) and *R* replication studies (*R*>5–6) on a smaller number of markers. The power calculation in each scenario was performed using the “Genetic Power Calculator” which is public available (http://pngu.mgh.harvard.edu/~purcell/gpc/). All the power calculations in the “Genetic Power Calculator” are based upon formula derived in Sham et al. [3].

##### Scenario one

Due to polygenic inheritance of complex diseases/traits, the chance of replicating a susceptibility variant is *much* lower than that of initially detecting it. This is because in a GWA study designed to identify quantitative trait loci (QTLs), it is usually easy to detect *one* of the QTLs, even if the effect of the detected QTL is small and the statistical power of the study is low. As a numerical demonstration, suppose there are 20 QTLs underlie a complex trait, each explaining 1% phenotypic variation. Assuming an ideal situation that the QTL effect size of 0.01, the QTL allele frequency of 0.20, the marker allele frequency of 0.30, linkage disequilibrium (LD) between the QTL and the marker of 0.80 (*D*′), and the significance level α = 5.0×10^−7^, a GWA study with a large sample of 4,000 unrelated subjects (commonly used in current GWA studies) may only have ∼10% power to detect a *specific* QTL among these 20 QTLs, but this GWA study has a much higher power of ∼88% (1−(1–10%)^20^) to detect *at least one* of the QTLs.

However, the probability of replicating *the specific* QTL detected in the initial GWA will generally be low in subsequent replication studies. For example, under the *same* ideal situation assumed above, an independent replication study with a sample size of 4,000 unrelated subjects will only have ∼49% power to replicate the initial finding at a much less stringent significance level of α = 10^−4^. Therefore, the *specific* QTL identified in the initial GWA can be replicated unless a much larger sample with much higher power is used. In practice it should be noted that the actual effect of the identified variant in an initial study is usually overestimated, a phenomenon called winner's curse [4]–[6]. Thus, the actual required sample size for replication is even larger than the one estimated based on the effect size reported in the initial discovery study.

##### Scenario two

We assume that two independent GWA studies are performed simultaneously. The likelihood that a *specific* susceptibility variant can be found by both studies (i.e., replicated by each other) is:
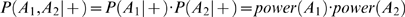
(1)where *P*(*A*_1_, *A*_2_|+), the probability of detecting the susceptibility variant by both studies, is the product of the power of each study [denoted as *power*(*A*_1_) and *power*(*A*_2_)]. When both studies have 10% power, the probability of detecting the same variant is as low as 1% (i.e., 10%×10%). This probability is increased to 64% when both studies have much higher power of 80%. However, when one study has a high power of 80% and the other has low power of 10%, the probability of detecting the same variant is still as low as 8% (10%×80%).

In the case of more than two independent GWA studies, consistent replication across studies becomes increasingly challenging and the probability of replication becomes diminishingly small unless each study has power close to 100%. Assuming that *N* independent GWA studies are performed, the probability of replicating a *specific* susceptibility variant by all the studies is
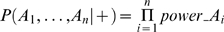
(2)


**Figure 1:**
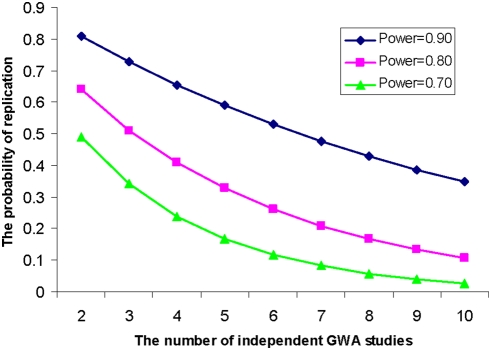
The probability of replication in different numbers of independent GWA studies. We assume all the studies have the same power (0.90, 0.80, and 0.70) to detect a specific genotype-phenotype association.

It can be seen that the likelihood of consistent replication by *N* individual studies depend on the statistical power of each study, and particularly, on the study of the *lowest* power. As the number of individual, independent studies increases, there is a dramatic decrease in the probability that the *same* variant will be consistently significant in all of those studies (shown in **Figure 1**).

A possible illustration for this scenario is four large, recently published GWA studies for type 2 diabetes [7]–[10]. The first published one was performed in a French case-control cohort [10]. The other three were performed, respectively, by the Finland-United States Investigation on NIDDM Genetics (FUSION) team [9], the Diabetes Genetics Initiative [8], and the Wellcome Trust Case-Control Consortium [7]. Each GWA identified a number of loci that may confer type 2 diabetes risk. However, only a limited few loci (e.g., *TCF7L2* and *SLC30A8*) showed significant associations across ALL the four studies. Notably, neither of these loci achieved a genome-wide significance level (a *P* value of 10^−7^ [11]) in all the four studies.

##### Scenario three

This scenario represents a hybrid of scenarios one and two. We assume that there are *N* independent GWA and *R* replication studies. Without loss of generality, let's consider a situation, where two independent GWA (*N* = 2) are followed by six replication studies (*R* = 6) on a smaller number of markers.

Suppose there are 20 QTLs, each explaining 1% phenotypic variance. As shown in Scenario one, under an ideal situation, a GWA study with a sample size of 4,000 may have only ∼10% power to detect *a specific* QTL among these 20 QTLs, but the study may have a high power of ∼88% (1–(1–10%)^20^) to detect *at least one* of these 20 QTLs. The probability that the same QTL can be identified by both initial GWA studies is only ∼8.8% (10%×88%). To achieve 80% power to replicate this association in follow-up studies (assuming *D′* = 0.80, QTL allele frequency of 0.20, marker allele frequency of 0.30, α level of 10^−4^, no between-study heterogeneity or other potential biases), a larger sample of at least 6,000 subjects is needed. Even if replication is achieved, there is still a possibility that the finding is false positive (shown below in section “Are Replicated GWA Findings Always True?”).

#### b) Influence of LD and allele frequency difference

GWA is an “indirect” approach testing association between markers and diseases/traits of interest. Thus, not only the frequency of susceptibility variants but also the markers may affect the likelihood of detecting/replicating associations. The influence of LD and allele frequency difference on association studies has been demonstrated for diseases [12]–[14], but their quantitative effects on *quantitative* traits (e.g., BMI) have not been studied. To further illustrate the principles for quantitative traits, we show how these population-specific characteristics (i.e., marker and susceptibility allele frequencies, and LD strength) may affect study power. We consider this in terms of marker effect size, which we can estimate in practical studies.

**Figure 2:**
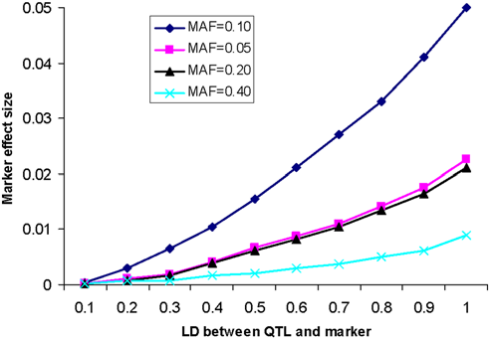
How marker effect size (y-axis) is determined by QTL effect size, marker allele frequency (MAF) and QTL allele frequency, and extent of LD between marker and QTL. We assume the QTL is under additive inheritance with MAF of 0.10 and effect size of 0.05 and the LD is measured by *D′* (x-axis).

Let's consider a diallelic QTL (*Q/q*) and a diallelic marker (*M/m*), with frequencies of *P_Q_*, *P_q_*, *P_M_*, and *P_m_*, respectively. Assuming LD (*D′*) between the QTL and the marker ranges from 0.1 to 1.0, we can calculate the marker effect size (see **Appendix S1** for detail). **Figure 2** shows how the measured marker effect sizes are influenced by frequencies of the QTL and marker and LD between them. It can be seen that the power of a GWA is greatest when allele frequencies of the QTL and the marker match. Discrepancies in allele frequency between the QTL and the marker may reduce power, often dramatically as the magnitude of this power reduction increases as the discrepancy in allele frequency increases. When susceptibility variant and marker allele frequencies match, the marker effect size is approximately proportional to the QTL effect size multiplied by the *D′* value between the two loci. Since allele frequencies of the QTLs and markers and LD strength may vary across populations [15]–[17], the power of GWA studies in different study populations may have different power to detect a QTL, even if the sample sizes are the same. A situation that may make replication even more difficult is long-range LD or cross-chromosome LD which may exist in the human genome [18]. Intuitively, such long range LD may have greater variation between different populations.

#### c) Confounding factors

Diversity in subject ascertainment and study design across studies may significantly impact GWA replication [11]. In addition, confounding factors, such as population stratification, genetic heterogeneity, environmental factors, and interactions between genetic and environmental factors (which may vary with different populations and environments), may reduce the chance of GWA replication. Most of these factors have been well recognized and evaluated in association studies for candidate genes [19]. Here we only briefly review these factors with regard to GWA studies.

Population stratification/admixture may mask, change or even result in apparently reversed genetic effect of genes underlying complex disease/traits. The available methods addressing/controlling this problem may have potential limitations [20]–[23]. For example, the Genome Control method [20] assumes that the degree of population differentiation is the same throughout the human genome, which may not the case. The performance of the Structured Association method [23] is highly dependent upon the amount and informativeness of ancestry informative markers (AIMs) [24], and the accuracy of inferred individual ancestry is sensitive to the number of pre-assigned subpopulations, which may not always be satisfactorily resolved [25]. In addition, the use of the Structured Association method for GWA studies is limited due to its intensive computational cost for large data sets. Principal component analysis [22] is a recently developed method specifically for GWA; however, it may lead to incomplete stratification correction when the number of markers is less than 20,000 [26].

Genetic heterogeneity is a common phenomenon in human diseases. A disease could be caused by different susceptibility variants in different populations. For a specific susceptibility variant, there may also exist genuine diversity of its genetic effect in different populations [27]. The allele frequency of a susceptibility variant could be different across different populations. Since the population genetic effect of a susceptibility variant depends on its allele frequency (**Figure 2**), it should not be surprising that a significant association identified in one population cannot be found in another. A recent study estimated the required sample size to replicate an association finding with different amounts of between-study heterogeneity [28]. The authors concluded that: 1) if between-study heterogeneity reaches certain thresholds, it may not be practically possible to consistently replicate some true associations, no matter how large the studies are, and 2) replication sample sizes of 40,000 subjects or even larger are essential for generating sufficient power to replicate an association of small or modest effect size [28].

Studies have shown that LD patterns vary substantially among different populations [29]–[32]. This differential LD (or LD heterogeneity) may cause nonreplication of GWA findings across studies in different populations[27]. LD heterogeneity may exist even in populations of the same ethnic group such as Caucasians of European ancestry [30], [33], [34], although data accumulated from recent GWA studies [35]–[37] tend to show that, between Caucasian populations, the LD is not very heterogeneous. The diverse intermarker LD may affect the probability of replicating a gene or region and this should be considered when designing a replication study, especially with regard to marker-selection strategy. Clarke et al. [38] showed that when a region of high intermarker LD is tested to replicate an initial finding that is only weak association with a disease, the “local” approach that involves both the originally significant markers and others in the same regions is a good strategy. Otherwise, the most powerful and efficient strategy for replication involves testing only the initially identified variants [38].

Populations of different or even similar cultures may have different exposures to environmental factors. For example, lifestyles such as diet, smoking, alcohol drinking, nutritional status, and exercises may have significant influence on human body fat (a focal phenotype for obesity research). These factors are sometimes difficult to be assessed and quantified accurately and their actual influence on body fat variation might not be judiciously accounted for in statistical analyses, although there is a debate on whether the statistical adjustment for environmental factors is necessary [39].

### 2) Are Replicated GWA Findings Always True?

In the field a common practice is that a significant GWA finding is considered to be “true” if replicated by several subsequent studies (not necessarily all). However, are replicated GWA findings always true? Some Bayesian based approaches (such as positive predictive value (*PPV*) to be discussed below) or complementary approaches (such as Bayesian False-Positive Report Probability) [40], [41] may help assess the credibility of association findings. In the following, we assess the probability of the replicated association being “true” using a Bayesian approach - *PPV* [42].

For an association finding, the *PPV* can be estimated by
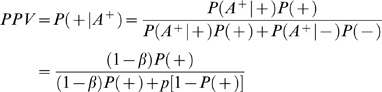
(3)where ‘+’ and ‘−’ denote the presence and absence of association, respectively. *A^+^* denotes rejection of the null hypothesis (i.e., a significant association is detected). *P*(*A^+^*|+) = 1−*β* is the statistical power and *P*(*A^+^*|−) = *p* is the type I error rate. *P*(+) is the prior (pre-study) probability that the association is true. Similarly, we can use negative predictive value (*NPV*) to evaluate the probability of null hypothesis being true (i.e., no association) when a follow-up study does not find the association. *NPV* can be estimated by
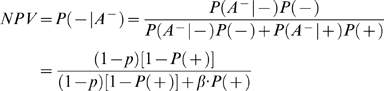
(4)where 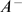 denotes acceptance of the null hypothesis (i.e., no association is detected).

Supposing a significant association is identified in the initial GWA, the probability of the identified association being true is
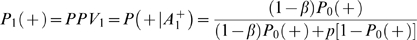
(5)where *P_1_*(+) is the *PPV* of the initial GWA.

Further suppose that *n* independent subsequent GWA studies are performed. If the *k*th study (*k*≤*n*) replicates the initial finding (i.e., the null hypothesis is rejected), the probability of the identified association being true is
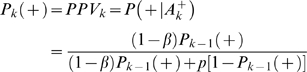
(6)


Alternatively, if the *k*th study does not replicate the initial finding (i.e., the null hypothesis is accepted), the probability the association being true is
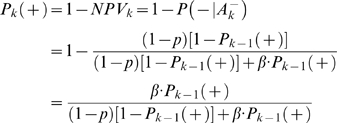
(7)


From Equations 5–7, it can be seen that the probability of a replicated GWA finding being true is determined by pre-study prior probability, statistical power, and *p* value of the test. Note, here the prior probability changes by incorporating the *PPV* of previous studies.

The above Equations are given without considering potential biases. Here biases refer to those factors (due to study design, data quality, statistical analysis, or result presentation) that tend to produce misleading research results. Let's quantify bias by *u* - the proportion of probed analyses that would not have been GWA findings in an unbiased study, but are nevertheless reported as such, because of bias [42]. By taking into account bias *u*, the probability of an identified association being true can be calculated as
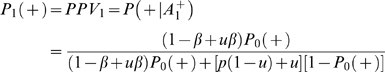
(8)


If *n* independent subsequent GWA studies are performed and the *k*th study (*k*≤*n*) replicates the initial finding, the probability of the association being true is
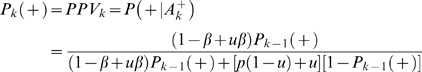
(9)


As a numerical illustration, we assume that a number of sequential GWA studies are performed using 500 K SNP arrays, among which 20 SNPs are susceptibility variants. Then the prior (pre-study) probability of finding *at lease one* of the susceptibility variants is *P_0_*(+) = 20/500,000 = 4.0×10^−5^, which is quite low compared to hypothesis-driven studies with prior knowledge or evidence of the tested association. We next consider an actual situation where both positive and negative results are involved. We assume that an initial GWA study identified a significant genotype-phenotype association, which is followed up by one to six subsequent independent studies. We show in **Table 1** the *PPV* values corresponding to various situations where follow-up studies replicated the initial finding. From Table 1, we can see that if three among four follow-up studies replicate the initial association and the fourth does not, the *PPV* of the identified association is only ∼0.31. This means the chance of being true is only 31% and the chance that this replicated finding is false is still as high as 69%. If assuming a bias *u* = 0.1, the *PPV* decreases dramatically to as low as 0.006. As another example, if four among six follow-up studies replicate an initial association and two other follow-up studies do not, the *PPV* of the identified association is only 0.46. While *PPV* increases with the increasing number of positive replication studies, each non-replicated study may dramatically decrease the *PPV* value. The above *PPV* calculation was based on an assumption that all the studies have high statistical power of 90%, which is often higher than that achieved in actual studies.

**Table 1:** The *PPVs* corresponding to various situations where different number of follow-up studies replicated the initial finding.

GWA studies		*PPV*						
Initial GWA		0.0007	0.0007	0.0007	0.0007	0.0007	0.0007	0.00072
Replication studies	1	0.012	**7.58E-05**	**7.58E-05**	**7.58E-05**	**7.58E-05**	**7.58E-05**	**7.58E-05**
	2	0.189	0.001	**7.98E-06**	**7.98E-06**	**7.98E-06**	**7.98E-06**	**7.98E-06**
	3	0.807	0.023	0.0001	**8.40E-07**	**8.40E-07**	**8.40E-07**	**8.40E-07**
	4	0.986	0.306	0.002	1.51E-05	**8.84E-08**	**8.84E-08**	**8.84E-08**
	5	0.999	0.888	0.044	0.0002	1.59E-06	**9.31E-09**	**9.31E-09**
	6	0.999	0.993	0.455	0.004	2.86E-05	1.67E-07	**9.8E-10**

*PPV*: positive predictive value.

Bold represents the follow-up study that does not replicate the initial finding.

It should be noted that the experiment-wise *α* level of 0.05 is used in the above *PPV* analysis. In GWA studies, to account for multiple comparison by testing hundreds of thousands SNPs, a point-wise *P* value of 10^−7^ for a genotype-phenotype association is considered to be statistically significant [11]. However, in *PPV* analysis, only the experiment-wise *P* values that are adjusted for multiple testing should be used. Otherwise, even point-wise *P* values on the order of 10^−2^–10^−5^ achieved in an initial GWA can yield high *PPV* if directly used in *PPV* analyses without correction for multiple testing.

## Discussion

### Integrating Evidence of Multiple Levels/Sources

By discussing the difficulties of replication, we do not intend to depreciate the GWA approach and subsequent replication efforts. This is an evolving area and our understanding of GWA is improving from the knowledge gained and challenges that remain. The genetic community has had some useful discussions on design, implementation, best practice and interpretation of GWA studies [11]. Even with the difficulty of replication notwithstanding, variants with relatively large effects may be identified and replicated in powerful GWA studies [11]. A potential practical example is the *FTO* gene, whose association with obesity and related phenotypes was identified in three independent GWA studies in French [35], German [36], and British populations [37]. This *FTO* gene is responsible for 1% of the total heritability of obesity [43]. Meta-analysis is a useful tool for synthesizing data and exploring potential heterogeneity [44], [45]. Joint (meta) analysis of data from comparable GWA studies may increase the power of gene identification when individual GWA studies are underpowered. However, meta-analysis may not always be ideal as it may suffer from potential problems such as between-study heterogeneity and bias (e.g., selective publication) [46].

From systems biology perspective, GWAs are studies at the DNA level. At best, GWA studies could, in conjunction with fine mapping efforts, identify the implicated variant(s) at the level of molecular markers (e.g., SNPs). Although GWA studies may implicate a gene(s) as a factor contributing to a disease/trait, it cannot tell anything about *how* the gene(s) contribute to the disease/trait. Similarly, bioinformatics tools, while useful, may only infer some of the functions of the identified markers/genes.

Functional genomic studies, including gene expression studies at the RNA level and proteomics studies at the protein level, may provide useful complementary information to GWA studies. For example, functional genomic studies may unravel critical information about the regulation of gene activity under various conditions that may contribute to our knowledge of molecular and genetic mechanisms influencing disease development. GWAs, gene expression and proteomics studies, individually, have shown some successes in identifying genes for complex diseases. However, each may be prone to false positive/negative findings, partially due to multiple testing in genomic approaches, statistical power (partially associated with conservative procedures accounting for multiple testing), and the biological complexity of gene expression and genetic etiology. Gene expression is regulated simultaneously and interactively at all the three levels, i.e., DNA, RNA, and protein levels. Gene expression may be regulated at the level of DNA, RNA, or protein and there is often interaction between regulatory controls at these different levels. Hence, a genomic convergence or systems biology based approach that integrates the information from GWA studies, gene expression and proteomics may facilitate the identification of key pathways that are globally involved in the pathogenesis of the disease and/or interactive factors acting at different levels of disease-gene expression [47]. It should be noted that genomic convergence may have its own limitations. Genes identified at the DNA level in GWA studies may escape confirmation/replication in studies of RNA (microarray) or protein (proteomics), partially because of inherent differences in these experimental approaches and complex regulation of gene functions involved, even when those genes actually contribute to disease development.

Gene expression levels can be used as quantitative traits in traditional linkage or association studies, and the identified loci are termed expression QTLs (eQTLs) [48]. Given that most common human diseases are outcome of a complex interaction between many genetic loci and the environment, there are obvious advantages to studying the genetics of gene expression in cells that represent the *in vivo* state. For example, a recent study analyzed the expression of 23,720 transcripts in large population-based blood and adipose tissue cohorts for various obesity related traits [49]. A core network module in humans and mice was identified that is enriched for genes involved in the inflammatory and immune response and was found to be causally associated to obesity-related traits [49].

Ultimately, the functional relevance of the identified variant(s) should be confirmed by *in vivo* or *in vitro* studies. These functional studies, however, also face challenges, as results of *in vivo* and *in vitro* studies might not be consistent, and findings obtained in animal models may not necessarily translate into those in humans. How to best integrate information of multiple levels/sources in gene identification and functional studies remain a topic for open discussion. This is an area that requires substantial efforts from biologists and clinicians, statistical geneticists, bioinformaticians, and epidemiologists.

In summary, identification of genes underlying human complex diseases is challenging. Replication, while important and valuable, is difficult to achieve and may not be sufficient or necessary for validating GWA findings. Additional information from other lines of evidence, such as detailed molecular mechanistic studies and genomic convergence, may be useful for validating and illuminating the functional relevance of genes identified in GWA studies.

## Methods

The power calculation was performed using the “Genetic Power Calculator” (http://pngu.mgh.harvard.edu/~purcell/gpc/). All the power calculations in the “Genetic Power Calculator” are based upon formula derived in Sham et al. [3].

In *PPV* analysis, the *PPV* values for various situations were calculated based on the Bayesian theory. The assumed parameters were under ideal situations.

## Supporting Information

Appendix S1 Determination of marker effect size by QTL effect size, allele frequency differences, and the LD between marker and QTL
(0.08 MB DOC)
