# Supplementary material for: Is Replication the Gold Standard for Validating Genome-Wide Association Findings?
Source: PLoS One. 2008 Dec 29;3(12):e4037. doi: 10.1371/journal.pone.0004037 (PMC2605260; doi:10.1371/journal.pone.0004037)
Supplement: Appendix S1 — Determination of marker effect size by QTL effect size, allele frequency differences, and the LD between marker and QTL (0.08 MB DOC) [file pone.0004037.s001.doc]

**Appendix S1**

**Determination of marker effect size by QTL effect size, allele frequency differences, and the LD between marker and QTL**

For simple demonstration, we consider a diallelic QTL (*Q/q*) and a diallelic marker locus (*M/m*), with allele frequencies denoted as *P_Q_, P_q_, P_M_*, and *P_m_*, respectively. There are a total of four haplotypes defined by the QTL and the marker: *MQ*, *Mq*, *mQ*, and *mq*, with haplotype frequency denoted as *P_MQ_*, *P_Mq_*, *P_mQ_*, and *P_mq_*. We assume that the linkage disequilibrium (LD) between the marker and the QTL, in terms of D', is from 0.1 to 1.0. The frequencies of the four haplotypes can be calculated as

$P_{MQ}=P_{M}P_{Q}+D$; $P_{Mq}=P_{M}P_{q}-D$; $P_{mQ}=P_{m}P_{Q}-D$; $P_{mq}=P_{m}P_{q}+D$ [A1]

For the diallelic QTL (*Q/q*), we denote the genotype value as *–a*, *ka*, *+a* for genotype *qq*, *Qq*, and *QQ*, respectively, where 2*a* represents the difference between the mean phenotypes of *qq* and *QQ*, and *k* provides a measuring of dominance. Allele *Q* and *q* behave in a completely additive fashion when *k*=0, whereas *k*=+1 implies complete dominance of the *q* allele, and *k*=-1 implies complete dominance of the *Q* allele. Then, for the nearby diallelic marker locus (*M/m*), genotypic value of *MM*, *mm*, and *Mm* can be calculated as

$$\mu_{MM}=\left\{aP_{M}\left[P_{M}\left(P_{Q}-P_{q}\right)+2D\right]+2ak\left[2P_{M}P_{m}P_{Q}P_{q}-\left(P_{Q}-P_{q}\right)\left(P_{M}-P_{m}\right)D-2D^{2}\right]\right\}/P_{M}^{2}$$

$$\mu_{mm}=\left\{aP_{m}\left[P_{m}\left(P_{Q}-P_{q}\right)-2D\right]+2ak\left[2P_{M}P_{m}P_{Q}P_{q}+\left(P_{Q}-P_{q}\right)\left(P_{M}-P_{m}\right)D+2D^{2}\right]\right\}/P_{m}^{2}$$

$\mu_{Mm}=\left\{2a\left[P_{M}P_{m}\left(P_{Q}-P_{q}\right)-D\left(P_{M}-P_{m}\right)\right]+2ak\left[2P_{M}P_{m}P_{Q}P_{q}+\left(P_{Q}-P_{q}\right)\left(P_{M}-P_{m}\right)D+2D^{2}\right]\right\}/2P_{M}P_{m}$ [A2]

And the population mean (*μ*) of the marker can be calculated as

$\mu=P_{M}^{2}\mu_{MM}+2P_{M}P_{m}\mu_{Mm}+P_{m}^{2}\mu_{mm}=4aP_{M}P_{m}\left(P_{Q}-P_{q}\right)+8kaP_{M}P_{m}P_{Q}P_{q}$ [A3]

Then the phenotypic variance (*V_Marker_*) attributable to the marker, i.e., the marker effect size, is

$V_{Mar\ker}=P_{M}^{2}\mu_{MM}^{2}+2P_{M}P_{m}\mu_{Mm}^{2}+P_{m}^{2}\mu_{mm}^{2}-\mu^{2}$ [A4]

Given the QTL effect size and allele frequencies of the QTL and marker, the marker effect size for any markers can be derived from the above formulas.
